# Special Analytical and Health Commission on Tinned and Preserved Food

**Published:** 1906-07-28

**Authors:** 


### SPECIAL ANALYTICAL AND HEALTH COMMISSION ON TINNED AND PRESERVED FOOD.

### V. CANNED MEATS.

From the domestic standpoint the importance of the class of foods represented by canned meats cannot be overestimated. As we pointed out in our last article, a diet must consist of three elements—fats, and carbohydrates supplying essentially the carbon of the diet, and proteids supplying the nitrogen. Carbohydrates and fats are to some extent interchangeable, and we can do without one or the other, but not both. This is, however, not true of the proteid or nitrogenous constituent of our diet, and although by increasing especially the carbohydrates of a diet we may diminish the proteids, we can never entirely dispense with them; in fact, speaking roughly, every working diet must contain about 120 grammes, or a quarter of a pound of proteid, or approximately 20 grammes of nitrogen per diem. The function of the proteid moiety of the diet is essentially to keep the framework and tissues of the body in repair, only a part of it being actually consumed or converted into biological energy. The dietetic source of proteid varies in different countries and under different social conditions. A tremendous change has occurred in the source from which the inhabitants of manufacturing towns have drawn the proteid of their diet during the last thirty years. During this time has occurred the birth and development of the preserved and frozen-meat trade. If it were not for these two sources of meat there would in this country be at once a meat famine. At the present time if we compare the price of meat in this country with that of meat in Germany, where the importation of meat is seriously hampered by protective legislation, we find the inhabitants pay just three-quarters of the price which is paid by the German. A rough estimate of the amount of meat consumed in this country is that it consists of slightly over a hundred pounds per annum per head, or roughly one-third of a pound per head per day. This consumption has increased very largely during the last three decades, and approximately 45.1 per cent. of the whole meat consumption is beef, and 30 per cent. pig-flesh. A very large quantity of the meat consumed consists of canned or preserved meat.

### Essential Features of Canning Process.

The essential feature in the canning of meat is the sterilisation of the original flesh by heat and the exclusion of air. A very large number of processes may be grouped under this head, but all are practically based on the process devised by Appert in 1809, in which the provisions were heated in earthenware vessels and protected from subsequent infection by being hermetically sealed. Cans are employed to a much greater extent now, owing to their greater strength and the readiness by which they can be made air-tight. In the large factories steam-retorts are used for sterilising, but in the smaller ones the cans are immersed in a water or salt bath. A small hole is left in the cover, and after the sterilisation, and while the can is still filled with steam, this is closed with a fragment of solder. Finally the cans are left for a week, and then each is tested by striking it on the head with a wooden hammer. If the cap sinks down slowly it is concluded the process of sterilisation has been properly carried out; but if, after being depressed, it springs back again and bulges it is termed a swell-head, and rejected. Corned beef is, of course, specially prepared, and is both pickled and sterilised. It is pickled in large vats, and, when salted, is cooked and packed by means of steam pressure into cans, which are immediately sealed. These sealed cans are put to stand from three to six hours in boiling water, and are then pierced, and water and fat allowed to escape. They are subsequently again soldered up and sterilised in a water bath.

### Properties of Good Canned Meat.

Having considered the essential features of the canning process, it is necessary to say something with regard to the properties of good canned or preserved meat as compared with bad. The can of good canned meat should not bulge—that is, neither the top nor the bottom should be convex, nor should its surface, top, bottom, or sides show what are called in the trade "blisters." When the tin is opened no gas should exude, nor should any gas be capable of being extracted by any of the apparatuses designed for this purpose. Any jelly surrounding the meat should be solid, and not liquid. The reaction of canned meat is usually acid, but it may be alkaline without necessarily being decomposed, especially in the case of fish. This is important, because metallic contamination of the tinned contents is more likely to occur when the reaction is alkaline. The essential difference between canned or preserved (sterilised) meat and the same meat sold by a butcher is that weight for weight the former is more profitable than the latter. If we take, for instance, beef, we find that ordinarily speaking fresh meat as sold by the butcher contains approximately 72 per cent. of water, 20 per cent. of proteid, 5 per cent. of fat, and 1.14 per cent. of ash. In the case of sterilised meat an average composition is the following: Water, 50 per cent.; fat, 10 per cent.; proteids, 29 per cent. Hence, there is, roughly speaking, 25 per cent. less water in canned meat than in fresh meat. A further difference is that in canned or preserved meat there is practically no waste material; bone, gristle, etc., having been removed.

### Preserved Beef.

We have received several samples of preserved beef. Messrs. Cosenza and Company, of 95 Wigmore Street, London, W., have submitted a tin of their cold beef with jelly. We regard this preparation as exceedingly good; both the beef and the jelly are of first-class quality, and possess a remarkably fresh and appetising taste. Hunter's Handy Ham Company have sent a 6-lb. tin of their best British beef. The tin as supplied to us is as this article is packed for export, but it can also be supplied in smaller quantities in cardboard boxes. This beef is guaranteed English, and we have no reason to doubt that is is composed of the best English beef. Our examination of it convinced us that this article, which is ready for the table, is made from the best material and is hygienically prepared. Messrs. Chivers and Sons, of Histon, Cambridge, have supplied a sample of their luncheon beef. This preparation is of high quality and excellent taste, and is put up in glass. It is a concentrated beef in that its water-content is exceedingly low; consequently it keeps quite long enough after being opened to fulfil ordinary domestic wants. We have received from Messrs. Joseph Travers and Sons, Limited, of 119 Cannon Street, a tin of Queensland corned beef (Q.M.E. brand). It is calculated that this beef is approximately equal to double its weight of uncooked beef, as it is both cooked and compressed. Our experience of this corned beef is that it is a perfectly good and wholesome article of diet, and in every way to be recommended. The Australian Meat Company have submitted a tin of their compressed corned beef (Ramornie brand). We understand that this Company supplies the Admiralty and War Office. The beef is prepared from the finest Australian cattle. When turned out it cuts well, is of good colour and texture, and it possesses an agreeable taste. It will be remembered from our former articles that this Company also make an extract of beef, which our examination enabled us to describe as a highly satisfactory product. The Liebig Extract of Meat Company have supplied us with a 2-lb. tin of their Fray Bentos corned beef. This beef is made from the prime cattle bred and reared on the Liebig Company's own cattle farms. The name of this Company will always be associated with pioneer work in the extract of meat and preserved meat industries. The name Fray Bentos recalls to all acquainted with the history of this important branch of dietetics the classical experiments of Pettenkoffer, Voit, and Justus von Liebig. This corned beef, the latest product of this Company, is quite up to their usual standard. The Sydney Meat Preserving Company have provided us with a 2-lb. tin of their compressed corned mutton. Mutton, from the dietetic standpoint, has certain advantages over beef, and we are glad to find that a preparation of this character is at the disposal of the public. The article in question is cooked and ready for the table, and has all the characteristics of good corned mutton.

### Invalid Preparations.

Since concluding our remarks upon soups, meat extracts, jellies, etc., we have received a number of Messrs. Brand and Company's invalid preparations. These are so well known, and have stood the test of so extensive a trial, that further comment upon them is almost needless. We note, however, that they now supply a veal broth for invalids of double strength, which we have used and find in every way satisfactory. They have also supplied us with a large tin of their fibrous beef-tea, designed expressly for hospital use. In our earlier article we discussed the nutritive advantage gained by adding to ordinary beef-tea the constituents of meat fibre. Messrs. Brand and Company have done this, and the result is eminently satisfactory. We also notice a distinct advance in technique in that Messrs. Brand and Company now put up their essences of beef and chicken in glass.

